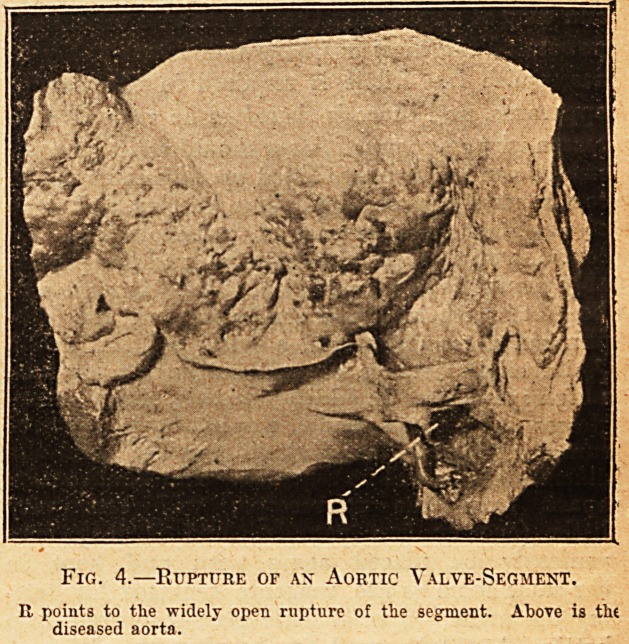# Some Affections of the Heart Connected with Sudden Death

**Published:** 1907-03-09

**Authors:** Theodore Fisher


### Hospital Clinics.

### SOME AFFECTIONS OF THE HEART CONNECTED WITH SUDDEN DEATH

By THEODORE FISHER, M.D., M.R.C.P.

### THE AORTIC VALVE.

The relation of diseases of the aortic valve to sudden cardiac failure is of a much more intimate character than is the case with disease of the mitral valve. There are, however, it is scarcely necessary to remark, varieties of diseases of the aortic valve, and the danger of sudden death is greater in some forms than in others. It is well known that aortic regurgitation is a more serious disease than aortic stenosis, but possibly it is not always realised that there are varieties of aortic regurgitation, and that the risk to life is not the same in every variety. Dr. Seymour Taylor has thought that in cases where there is also mitral regurgitation the outlook is less serious than when disease is limited to the aortic valve, owing to the fact that dangerous over-distension of the left ventricle is relieved by escape of blood through the mitral orifice. That may be so, but there are other reasons why one form of aortic regurgitation is more dangerous than another.

Before indicating these reasons it may be interesting briefly to refer to various forms of disease of the valve. In the first place we have the results of rheumatic endocarditis, secondly there is aortic valve disease associated with disease of the aorta, and thirdly, a calcification of the segments of the valve occurs which apparently is quite a distinct disease from atheroma of the aorta, and is most commonly present where there is no disease of this vessel.

Here it may be mentioned, however, that calcification is not confined to one form of endocarditis. Lime salts may be deposited in any chronic inflammation of the endocardium. In old-standing rheumatic disease, whether of the aortic or mitral valve, the presence of calcareous masses is not uncommon, yet there is a disease of the aortic valve in which early calcification is the most prominent feature. This disease sometimes leads to the formation of very large masses of calcareous material, which, it is needless to say, greatly interfere with the outflow of the blood from the ventricle. And even when these masses are not large, the segments of the valve may, by the deposit of salts, be rendered so rigid that they will only allow a very small stream to pass between them. This disease, it is scarcely necessary to remark, causes aortic stenosis. It has been said that aortic stenosis unassociated with aortic regurgitation is extremely rare. The experience of most observers no doubt varies considerably, but it has happened that in the course of my post-mortem work pure aortic stenosis has not been very rare—at least I have met with nine cases in which there was no evidence of regurgitation. Fig. 1 is a photograph from an example which occurred in a man aged 55, who had cut his throat on account of urgent dyspnœa. It illustrates, what curiously enough appears to be not uncommon in these cases of calcareous disease, that the aortic valve is composed of only two segments. The liability of the aortic valve when composed of two, instead of three, segments, to suffer from calcareous disease has attracted the attention of Dr. Parkes Weber and other observers. An interesting fea-

Fig. 1.—Calcareous Disease of the Aortic Valve.

The valve is viewed from above. There are only two valve-segments. The letter A, placed near the cut margin of the aorta, points to calcareous masses in these segments.

ture of this disease, also worthy of notice, is that, *although it is often spoken of as atheroma, the aorta is almost invariably healthy.* Not much of the aorta can be seen in fig. 1, yet there is possibly sufficient to show that no calcareous degeneration is present in this vessel. Absence of disease of the aorta is more clearly seen in fig. 2, which is given because it illustrates another feature of calcareous disease of the aortic valve. A calcareous process is seen extending downwards into the large flap of the mitral valve. Instead of extending into the mitral valve a similar process, or processes, may invade the wall of the heart; there is, in fact, a small process invading the septum ventriculorum in this case. This local extension suggests that the disease is of a locally infective character, and if locally infective it is strange that the aorta almost invariably remains healthy. This seems to be especially noteworthy because another variety of aortic valvular disease frequently appears to owe its existence to neighbouring disease in the aorta. Before leaving calcareous disease of the aortic valve, it should be mentioned that this variety is most frequently met with after middle life, yet I have seen a well-marked example as early as the age of 27.

The most common cause of aortic stenosis is calcareous disease, but in rheumatic endocarditis also the segments of the valve may occasionally become adherent and so produce stenosis though the valve remains competent. This, however, it is needless to say, is rare, because the associated fibroid thickening of the segments almost invariably causes them to shrink, and thus renders them incapable of meeting at the central point of contact. I could, however, give two illustrations of aortic stenosis un-

FIG. 2.—CALCAREOUS DISEASE OF THE AORTIC VALVE.

A is placed near the junction of two adherent and calcareous valve-segments. P on a calcareous process extending downwards into the large flap of the mitral valve as far as its apex. S close to another smaller calcareous process entering the septum ventriculorum. It will be noticed that the aorta is healthy.

complicated by regurgitation and due to disease of the valve following rheumatism.

Aortic regurgitation due to shrinkage of the valve-segments following rheumatism scarcely requires comment, but it may be of interest to give brief attention to another and important variety of regurgitation through the aortic orifice. This variety is that which is associated with disease of the aorta. Occasionally disease of the aorta may render the aortic valve incompetent by causing dilatation of the aortic orifice while the segments of the valve remain healthy; but more commonly these segments are thickened and shrunken. This thickening is not due to the presence of calcareous changes, but to the contraction of fibrous tissue. A feature, therefore, which I should like to emphasise is that *in disease of the aortic valve-segments associated with disease of the aorta fibroid thickening and consequent shrinking of segments is the main feature.* Deposition of calcareous salts may occur, but it is very small in amount compared with what may be seen in calcareous disease of the aortic valve-segments where the aorta is healthy.

Here a remark may be made concerning disease of the aorta itself. While fatty degeneration, with subsequent deposition of calcareous salts, is a characteristic feature of the disease called atheroma, there is a variety of disease of the aorta in which areas of greyish thickening, instead of yellow thickening, are present. Here and there may be small yellowish white patches and streaks on the summits of the grey areas, but they affect only a small part of the diseased vessel-wall. It is with this variety of disease of the aorta that disease of the aortic valve-segments is most commonly associated. Fig. 3 gives some idea of this variety of disease of the aorta, and the thickened valve-segments are seen below. Aortic valvular disease associated with disease of the aorta is most commonly met with in middle life, but at a rather earlier age than the calcareous variety.

It is with this form of aortic valvular disease that sudden death most commonly occurs. Yet a fact must be mentioned that may at first sight appear strange. Although aortic regurgitation in which disease of the first part of the ascending arch of the aorta is also present is the most serious variety of aortic valvular disease, cases of sudden death are very commonly met with where disease of the aorta in this situation exists while the aortic valve-segments remain healthy and competent. A word of explanation may be necessary. Sudden death in aortic valvular disease has been frequently attributed solely to mechanical causes consequent upon leakage of the valve. Under conditions of extra strain the over-filled left ventricle has been thought permanently to cease to act in the midst of diastole, being unable to discharge contents which are unusually excessive in amount. Such may be the explanation in some cases, but most commonly it is not over-filling of the ventricle, but the condition of the cardiac muscle which is responsible for the abrupt arrest of cardiac action. The reason is as follows: The disease of the aorta frequently interferes with the circulation through the heart-wall by obstructing the orifices of the coronary arteries, and thus causing degenera-

FIG. 3.—DISEASE OF THE AORTIC VALVE ASSOCIATED WITH DISEASE OF THE AORTA.

The aortic valve-segments are thickened and rounded. Above them is the extensively diseased aorta.

tion of the cardiac muscle. In cutting into the heart-wall in such a case of sudden death, the muscle will be seen to be studded with patches of fibrous tissue of various sizes, and microscopical examination generally shows also the presence of fatty changes. A point of interest and importance is, therefore, that *the serious nature of aortic valvular disease associated with disease of the aorta is largely due to the fact that the disease of the aorta obstructs the orifices of the coronary arteries. As a consequence of this obstruction, the nutrition of the cardiac muscle suffers, fatty and fibroid changes occur, and there is thus a more serious menace to life than any variety of valvular disease in the shape of disease of the cardiac muscle.*

Disease of the cardiac muscle found in association with aortic valvular disease does not, however, occur only when disease of the aorta is present. It was long ago suggested that disease of the aortic valve may interfere with the circulation through the coronary arteries. At least one of these ideas has been shown to be erroneous; yet my experience would lead me to believe that the cardiac muscle presents serious morbid changes much more often in aortic valvular disease—whether the form be mainly that of stenosis or of regurgitation—than in diseases of the mitral valve. I refer now to cases of aortic valvular disease where the aorta is healthy. As an example, the case of a young man, aged 18, who fell dead while standing by the side of a horse may be mentioned. Thickening and adhesion of the aortic valve-segments were present and on cutting into the heart muscle extensive fibrosis was everywhere to be seen.

At the age of 18 any other cause for chronic valvular disease than rheumatism is rare, and the fibrosis of the cardiac muscle may have been a sequence of rheumatic myocarditis. Yet I have seen similar fibrosis in the heart of a middle-aged virgin, where aortic stenosis of the calcareous type was present. I use the word " virgin " because the condition it describes virtually excluded the possibility of syphilis having been the explanation of the condition of the cardiac wall, and the ordinary causes for fibrosis of the cardiac muscle were also absent. It seemed probable, therefore, that the heart-wall must have suffered in consequence of defective nutrition dependent upon stenosis of the aortic valve. But whatever the cause of degeneration of the cardiac muscle in aortic valvular disease may be, it is this degeneration of the muscle which is most to be feared when the question of sudden death is being considered.

Although such an accident as rupture of an aortic valve-segment has not, as far as I am aware, been recorded as a cause of sudden death, it may rapidly lead to a fatal issue, and a few words upon the condition may not be out of place. It has been thought that such an accident may occur in a perfectly healthy valve. That seems to me to be improbable. In most of the examples seen in museums, and also in the majority of recorded cases, there has been disease of the aorta. This disease of the aorta appears to have been of the character already referred to as the grey variety that is frequently associated with aortic regurgitation due to disease of the valve. Usually, as has been mentioned, the disease of the valve-segments is a fibroid thickening which leads to their shrinkage. Instead, however, of becoming thickened and contracted, a valve-segment may bulge and rupture, or may become torn away from some portion of its attachment to the aorta. If we allow the disease of the aortic valve to be of the same nature as that of the aorta, the occasional presence of yielding and rupture is not surprising when we consider how commonly the disease of the aorta occasions aneurysm. Fig. 4 is from a photograph of a specimen showing rupture of an aortic valve-segment. Above the valve is seen puckering and thickening of the aorta due to disease. The specimen is from a man, aged 40, who was seized suddenly with dyspnœa; but death did not occur until four months later. Another accident, somewhat similar to rupture and occurring also in association with disease of the aorta, is retroversion of an aortic valve-segment. In one case which came under my notice the retroversion occurred in a travelling acrobat, who obtained his living by turning somersaults in the air and performing similar feats which require considerable effort. In his case the retroversion of an aortic valve-segment, found after death, proved to be associated with disease of the first part of the aorta.

FIG. 4.—RUPTURE OF AN AORTIC VALVE-SEGMENT.

R points to the widely open rupture of the segment. Above is the diseased aorta.

In closing these remarks upon diseases of the aortic valve and their relation to sudden death, a few points may be recapitulated.

Sudden death may, as is well known, occur in any variety of aortic regurgitation, and less commonly in aortic stenosis.

The form of aortic regurgitation in which sudden death is most common is that in which there is also disease of the first part of the ascending aorta, and the abrupt arrest of cardiac action is mainly due to disease of the heart-wall.

In other cases of aortic valvular disease in which sudden death occurs, whether the variety be of the calcareous type, or whether it be that which follows rheumatism, fibrosis of the cardiac muscle will probably be present.

## Figures and Tables

**Fig. 1. f1:**
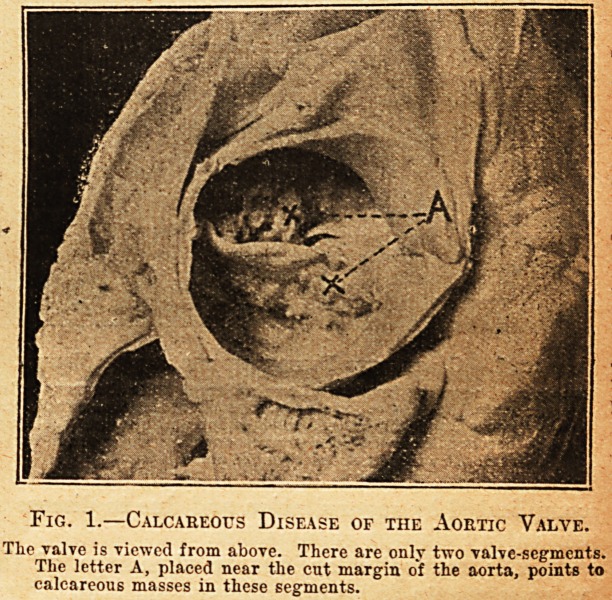


**Fig. 2. f2:**
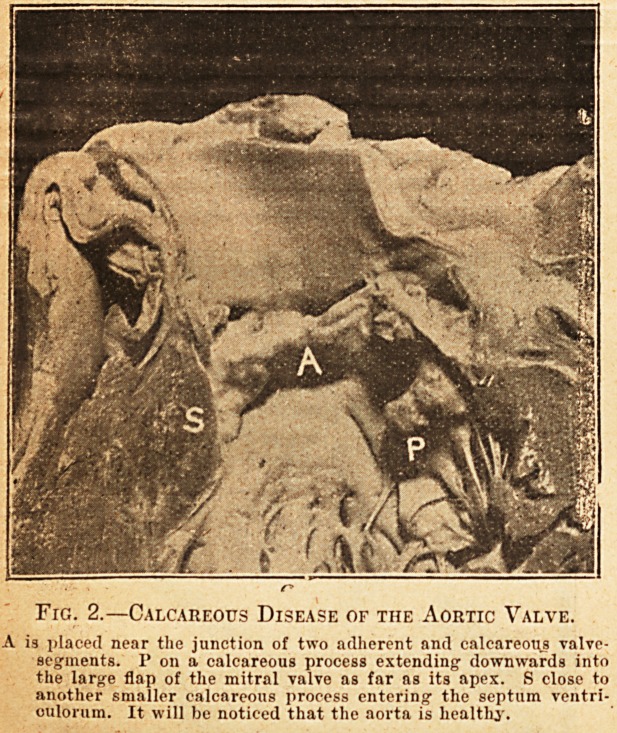


**Fig. 3. f3:**
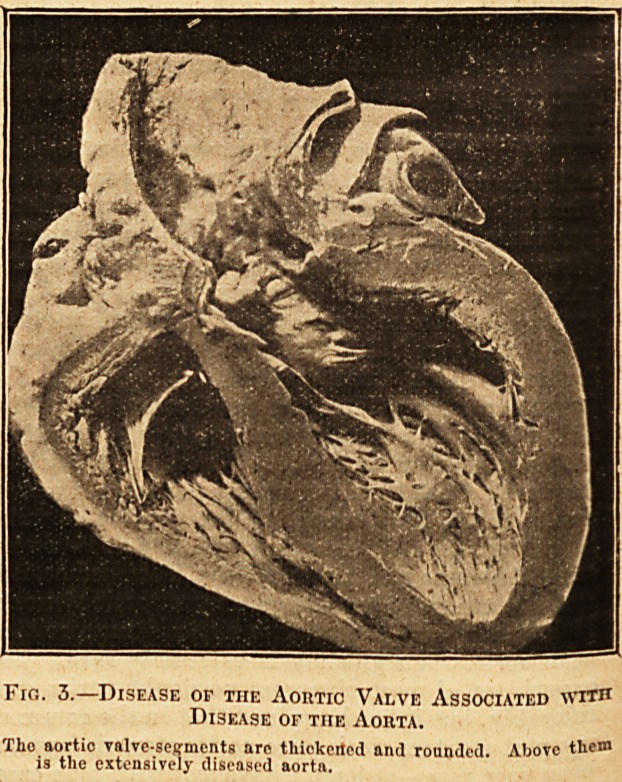


**Fig. 4. f4:**